# First Insights into the Subterranean Crustacean Bathynellacea Transcriptome: Transcriptionally Reduced Opsin Repertoire and Evidence of Conserved Homeostasis Regulatory Mechanisms

**DOI:** 10.1371/journal.pone.0170424

**Published:** 2017-01-20

**Authors:** Bo-Mi Kim, Seunghyun Kang, Do-Hwan Ahn, Jin-Hyoung Kim, Inhye Ahn, Chi-Woo Lee, Joo-Lae Cho, Gi-Sik Min, Hyun Park

**Affiliations:** 1 Unit of Polar Genomics, Korea Polar Research Institute, Incheon, South Korea; 2 Polar Sciences, University of Science & Technology, Yuseong-gu, Daejeon, South Korea; 3 Department of Biological Sciences, Inha University, Incheon, South Korea; 4 Nakdonggang National Institute of Biological Resources, Sangju, South Korea; Xiamen University, CHINA

## Abstract

Bathynellacea (Crustacea, Syncarida, Parabathynellidae) are subterranean aquatic crustaceans that typically inhabit freshwater interstitial spaces (e.g., groundwater) and are occasionally found in caves and even hot springs. In this study, we sequenced the whole transcriptome of *Allobathynella bangokensis* using RNA-seq. *De novo* sequence assembly produced 74,866 contigs including 28,934 BLAST hits. Overall, the gene sequences were most similar to those of the waterflea *Daphnia pulex*. In the *A*. *bangokensis* transcriptome, no opsin or related sequences were identified, and no contig aligned to the crustacean visual opsins and non-visual opsins (i.e. arthropsins, peropsins, and melaopsins), suggesting potential regressive adaptation to the dark environment. However, *A*. *bangokensis* expressed conserved gene family sets, such as heat shock proteins and those related to key innate immunity pathways and antioxidant defense systems, at the transcriptional level, suggesting that this species has evolved adaptations involving molecular mechanisms of homeostasis. The transcriptomic information of *A*. *bangokensis* will be useful for investigating molecular adaptations and response mechanisms to subterranean environmental conditions.

## Introduction

Subterranean fauna form below the surface of the earth. Hyporheic/groundwater environments are harsh for animals due to limited space, permanent darkness, low dissolved oxygen concentrations, and limited energy/food inputs. The environment includes two major ecosystems, namely stygofauna (aquatic and living in groundwater) and troglofauna (air-breathing and living in caves and voids) [1]. Particularly, subterranean fauna exhibit several ecological and physiological characteristics that are evolutionary adaptations to the extreme environmental conditions. These adaptations include a high tolerance to hypoxia, low metabolic rates, longevity, delayed maturity, smaller clutch size, and simple food webs with few trophic links [2, 3]. Also, many hyporheic/groundwater organisms show phenotypical or morphological convergence such as reduced pigment, poorly functioning eyes or eye loss, development of non-optic sensory organs, and/or relative lengthening of appendages [4]. Although species richness is relatively restricted in the subterranean fauna, groundwater habitats develop unique biodiversity [5]. The groundwater ecosystem is composed mainly of tiny crustaceans, oligochaetes, nematodes, acari, and molluscs that have small body sizes of < 1mm to several centimeters [6]. Due to limited distribution, poor competitive ability, and low reproduction, the hyporheic/groundwater ecosystem is particularly vulnerable to environmental stressors and anthropogenic contamination.

Crustaceans such as amphipods, isopods, copepods, and bathynellaceans are most dominant animal groups found in most groundwater habitats [7]. Almost all major taxonomic groups of crustaceans in groundwater also occur in surface water. Bathynellaceans, however, have been known to have exclusively occurred only in groundwater since the Palaeozoic. Bathynellacea (Crustacea, Syncarida, Parabathynellidae), are widely distributed in most parts of the world except Antarctica, but its species have been poorly studied due to the interstitial aquatic environments they inhabit [8, 9]. The genus *Allobathynella* Morimoto and Miura, 1957 has been characterized mostly in Eastern Asia (e.g., Japan and South Korea) and includes many species that were formerly assigned to *Parabathynella* [10]. Recently, 14 new species of *Allobathynella* were identified and characterized in South Korea [11]. Although molecular tools, such as allozyme and mitochondrial DNA analyses, have been successfully applied to investigate endemism, cryptic species, and the distribution patterns of subterranean syncarids [12], little is known regarding gene sequences, expression, and molecular evolution.

Here, we present the first report on the transcriptome of the subterranean crustacean *Allobathynella bangokensis* Park and Cho, 2016 including analyses of basal-level mRNA expression. We analyzed distinct or conserved gene families in *A*. *bangokensis* in comparison with the transcriptional profiles of several crustacean species. This information provides a whole transcriptomic dataset that will help our understanding of the molecular characteristics of subterranean syncarids.

## Materials and Methods

### Allobathynella bangokensis

*Allobathynella bangokensis* was collected from the subterranean region of Hongcheon-Gun, Gangwon-Do, South-Korea (37° 41‘N, 127° 40‘E). Species identification was confirmed by assessing its morphological characteristics using stereomicroscopy and mitochondrial cytochrome oxidase subunit 1 (CO1) sequence analysis. Collected *A*. *bangokensis* specimens were stored immediately at −80°C for subsequent RNA extraction.

### Ethics statement

No specific permits were required for the described field studies: a) no specific permissions were required for these locations/activities; b) location are not privately-owned or protected; c) the field studies did not involve endangered or protected species.

### RNA extraction and library construction

Total RNA was extracted using the the RNeasy^®^ Micro Kit (Qiagen) and the RNase-Free DNase I Kit (Qiagen, Valencia, CA, USA) according to manufacturers’ instructions. Whole bodies of 15 adult *A*. *bangokensis* were pooled and homogenized in RLT buffer (Qiagen). Extracted RNA was stored in RNA stable^®^ (Biometrica, San Diego, CA, USA) to prevent RNA degradation during long-term storage. Extracted RNA quality and concentration were determined using the 2100 Bioanalyzer (Agilent Technologies, Santa Clara, CA, USA). A next-generation sequencing (NGS) library was constructed from 2 μg total RNA using NuGEN Encore^®^ Complete RNA-Seq Library Systems (NuGEN, San Carlos, CA, USA). Final transcriptomic library lengths and concentrations were determined using the 2100 Bioanalyzer. Transcriptomic libraries were sequenced by the MiSeq^®^ System (Illumina) platform using sequenced runs of 300×2 paired-end reads. Index and adaptor sequences were trimmed using Trimmomatic [13] and low quality reads were removed using the FASTX tool kit [14].

### De novo assembly and annotation

We performed *de novo* assembly using software packages designed for short read sequence assembly, including Abyss [15], Velvet [16], CLC Genomics Workbench 7.5 environment (CLC Bio Aarhus, Denmark), and Oases (D.R. Zerbino, European Bioinformatics Institute). We assembled each sample using the same assembly parameters (K-mer length = 27, coverage cutoff = 10, minimum contig length = 200 bp). Consideration of the assembly statistics (*N50*, longest contig, number of contigs, proportion of reads assembled) led us to finally choose Oases, which generated the longest assembled ESTs.

### Data deposition

The raw sequencing reads of *A*. *bangokensis* were deposited in the Sequence Read Archive in GenBank (SAMN05712658).

### Annotation and gene ontology (GO) analysis

GO and Kyoto Encyclopedia of Genes and Genomes (KEGG) pathway analyses of the contigs were performed using the Blast2GO sequence annotation tool [17]. The three main categories biological processes, cellular components, and molecular functions were obtained after aligning contigs using default parameters. The assembled data were arranged including read length, gene annotation, GenBank number, E-value, species, and the species accession number. The assembled data, including GO terms, were deposited as supplementary material (S3–S5 Tables). In each section, the specific GO term composition was calculated and presented as a percentage.

### Gene expression analysis

The gene expression level of the *A*. *bangokensis* transcriptome was calculated using the reads per kilobase transcriptome per million mapped reads (RPKM) method [18]. Heat map analysis was conducted to represent the transcriptomic profiles using MeV software (ver. 7.4; Dana-Farber Cancer Institute, Boston, MA, USA).

## Results and Discussion

### Transcriptome assembly and gene annotation

To establish the transcriptomic database of Bathynellacea, we performed RNA-seq using the subterranean crustacean *Allobathynella bangokensis*. After trimming and assembly, a total of 63 Mb including 74,866 contigs of *A*. *bangokensis* was obtained by Illumina sequencing (Table 1). The lengths of the *A*. *bangokensis* contigs ranged from 200 to 26,238 bp, with an average length of 843 bp, and the N50 values of those contigs were 1,302 bp. To our knowledge, this is the first whole transcriptome study conducted in Bathynellacea.

**Table 1:** Summary of the statistics from the transcriptomic analysis of the subterranean crustacean *Allobathynella bangokensis*.

**Raw data**	
Reads no.	16,865,850
Reads length (bp)	4,010,699,130
**Raw sequence after QC**	
Reads no.	15,954,043
Reads length (bp)	3,703,731,082
***De novo* assembly**	
Contigs no.	74,866
Contig length (bp)	63,112,330
Length distribution (bp)	200 to 26,238
Average length (bp)	843
N50 (bp)	1,302

**Fig 1:**
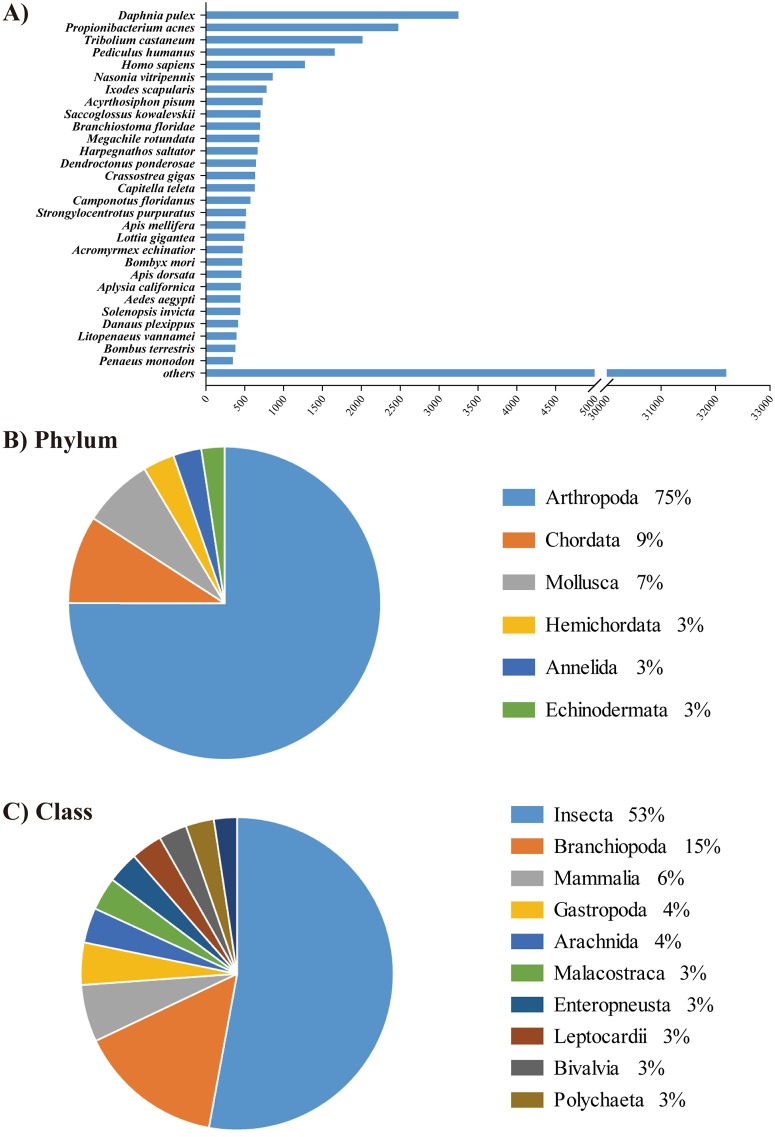
BLAST top hit species distribution. Number of top BLAST hit species identified for the *Allobathynella bangokensis* transcript contigs (A) and pie charts presenting the annotated genes and their corresponding phylum (B) and class (C). Detailed information is appended in the supplementary materials (S2 Table).

Gene annotation of the transcripts was performed by BLASTx analysis using the NCBI non-redundant (NR) protein database. The results showed that 28,934 contigs (38.6%) had at least one positive BLAST hit (E-value < 1e-04) representing 11,751 annotated genes (S1 Table). Distribution analysis showed that 18 species had more than 500 BLAST transcript hits, and the waterflea *Daphnia pulex* (Crustacea, Branchiopoda) showed the highest similarity with 3,250 reads (S2 Table; Fig 1A). Among the top hit species, 75% of contigs matched sequences of the phylum Arthropoda, while the other 25% was comprised of Chordata, Mollusca, Hemichordata, Annelida, and Echinodermata (Fig 1B). In addition, 53% and 15% of contigs showed homologies to insects and crustaceans (Branchiopoda), respectively, at the class level (Fig 1C). In the NCBI NR database, a relatively a huge amount of gene information of insects was appended compared to those of crustaceans. Thus, although Bathynellacean is an order of crustaceans, the overall gene annotation results represent high quality of assembled transcripts of *A*. *bangokensis* [19].

### Functional annotation

InterProScan protein sequence analysis and classification can be used to effectively classify protein functions by predicting domains and important sites [20]. The most abundant InterPro domains are presented in Table 2 (S1 Fig). The InterPro domains with the highest numbers of hits were immunoglobulin (IG)-like domain (IPR007110; 2433 hits), followed by P-loop containing nucleoside triphosphate hydrolase (IPR027417; 2342 hits) and fibronectin type III (IPR003961; 1761 hits). The immunoglobulin superfamily (IgSF) is a large group of soluble cell surface proteins that are mostly involved in adaptive immune defenses (e.g., recognition, binding, or adhesion processes), which are believed to be restricted to vertebrates [21]. All members of IgSF possess at least one IG-like domain or IG fold. The basic molecular mechanisms of the IG-like domain have rarely been investigated in invertebrates, but several genes possessing IG domains such as the C-type lectin domain, Down syndrome cell adhesion molecules, fibrinogen-related proteins, and hemolin have been suggested to be involved in host defense mechanisms of arthropods including crustaceans and mollusks [22–28]. Based on the relationship between IgSF and invertebrate immunity, it is possible that *A*. *bangokensis* has a robust innate immune defense system involving IG-like domain-containing proteins.

**Table 2:** The most abundant InterPro domain classifications of *Allobathynella bangokensis* transcript contigs.

InterPro ID	Domain description	Number of matched contigs
IPR007110	Immunoglobulin-like domain	2,433
IPR027417	P-loop containing nucleoside triphosphate hydrolase	2,342
IPR003961	Fibronectin type III	1,761
IPR013783	Immunoglobulin-like fold	1,583
IPR007087	Zinc finger, C2H2	1,491
IPR002172	Low-density lipoprotein (LDL) receptor class A repeat	1,327
IPR001478	PDZ domain	1,085
IPR000719	Protein kinase domain	1,040
IPR002110	Ankyrin repeat	997
IPR020683	Ankyrin repeat-containing domain	986
IPR001680	WD40 repeat	864
IPR001611	Leucine-rich repeat	820
IPR013087	Zinc finger C2H2-type/integrase DNA-binding domain	809
IPR000504	RNA recognition motif domain	773
IPR012677	Nucleotide-binding alpha-beta plait domain	742
IPR001452	SH3 domain	687
IPR001781	Zinc finger, LIM-type	685
IPR000742	EGF-like domain	650
IPR002126	Cadherin	647
IPR000859	CUB domain	641

**Fig 2:**
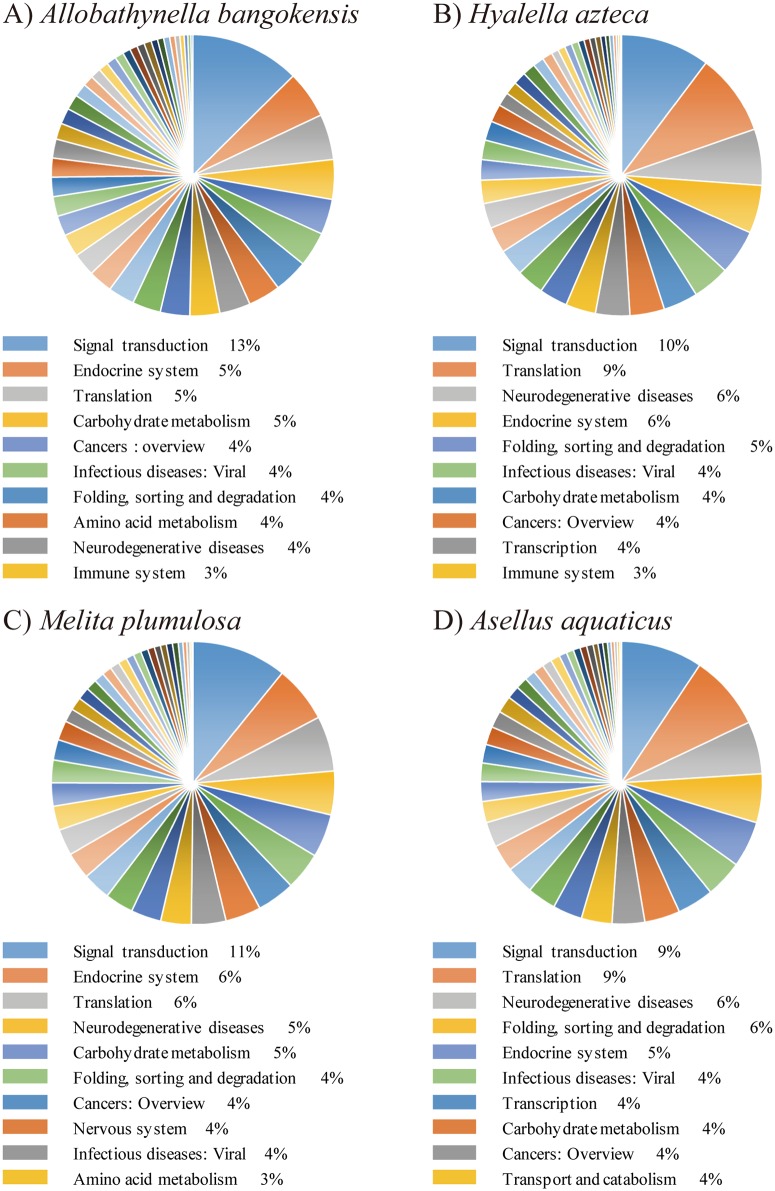
The composition of Malacostraca KEGG analysis. The compositions and percentage rankings of the A) *Allobathynella bangokensis*, B) amphipod *Hyalella azteca*, C) amphipod *Melita plumulosa*, and D) isopod *Asellus aquaticus* KEGG pathways. Detailed information is appended in the supplementary materials (S6 Table).

GO terms related to the top domains were described at the second level (S2 Fig). Detailed GO distributions in three GO categories (biological process, cellular component, and molecular function) are shown in the supplementary material (S3–S5 Tables). In the biological process category, many genes are classified as metabolic processes (28%), cellular processes (25%), and single-organism processes (19%). In the molecular function category, the vast majority of genes are involved in catalytic activities (43%) and binding functions (40%). In the cellular component class, most genes are related to the cell (36%) and cell membrane (24%). The overall proportions of the major GO categories in *A*. *bangokensis* sequences were very similar to those of the transcriptomes of several other crustaceans [29–32], and we were not able to identify significant differences in the proportions of the three GO term categories. KEGG analysis of the *A*. *bangokensis* transcriptome revealed that the vast majority of KEGG pathways are involved in signal transduction (13%), followed by the endocrine system (5%), translation (5%), and carbohydrate metabolism (5%) (Fig 2A; S6 Table). The composition rate and percentage rankings of the top 10 *A*. *bangokensis* KEGG pathways were similar to those of amphipods [30, 33] (Fig 2B; *Hyalella azteca* and Fig 2C; *Melita plumulosa*) and an isopod [34] (Fig 2D; *Asellus aquaticus*). Thus, these results suggest the intactness of the *A*. *bangokensis* transcriptome, since the information does not lack major functional GO categories or KEGG pathways compared with the transcriptomes of arthropods.

### Opsin repertoire

Opsins have been classified into four major monophyletic groups: 1) ciliary photoreceptors (‘c-opsins’), 2) rhabdomeric photoreceptors (‘r-opsins’), 3) cnidarian opsins (‘Cnidops’), and 4) a mixed group consisting of ‘retinal G-protein coupled receptors’, peropsins and neuropsins [35]. Interestingly, in the *A*. *bangokensis* transcriptome, we were not able to identify an opsin contig. Of the 28,934 Blast hit results, several candidates identified as putative opsin or relevant genes were searched, but our additional analysis using an in-depth annotation platform (e.g., domain analysis, phylogenetic analysis) revealed that the sequences were named incorrectly, because the contigs were matched to previous incorrectly registered opsin genes of other species in the NR database. In addition, short partial contigs of *A*. *bangokensis* that showed low similarity in various regions of the amino acid sequence could not be annotated to an opsin gene. To investigate putative opsin transcripts in *A*. *bangokensis*, a total of 50 opsin amino acid sequences from arthropods and onychophorans (numbered by 1–50 in S7 Table; i.e., long wavelength sensitive; middle wavelength sensitive; short wavelength sensitive groups, Blue and UV; onychopsin) annotated in a previous report [36], 7 amphipod opsins (numbered by 51–57 in S7 Table), and 21 arthropsins (numbered by 58–78 in S7 Table) and 25 peropsins (numbered by 79–103 in S7 Table) of arthropods, and additional 8 invertebrate melanopsins (numbered by 104–111 in S7 Table) registered in GenBank were mapped directly to 74,866 contigs of *A*. *bangokensis* using an internal local BLASTx platform. However, no contig of *A*. *bangokensis* was aligned to the gene pool comprised of opsin, arthropsin, peropsin, and eye pigmentation genes. In addition, we attempted to identify several eye-related genes previously annotated in the crustacean lineage [37]. Although these genes are involved in diverse functions beyond eye development or vision, many were transcriptionally not detected in the *A*. *bangokensis* (Table 3). Expression of the Dachshund (*Dac*) gene is important for controlling cell fate determination in eye, limb, brain, and muscle development [38]. *Drosophila* eyes absent (Eya) plays an essential role in retinal cell survival and differentiation [39], while multiple functions of Eya homologues (Eya1, Eya2, Eya3, and Eya4) have been identified continuously in mammals [40]. These two genes, *Dac* and *Eya*, act synergistically to induce ectopic retinal development and positively regulate each other’s expression through conserved domains in *Drosophila* [41]. Eyegone, which is a member of the *Pax* transcription factor family, was discovered for its essential requirement in retinal primordium growth in *Drosophila* [42, 43]. We were unable to determine the transcriptional roles of these genes in *A*. *bangokensis*, but overall transcriptional patterns that are not detected in the transcriptome would affect phenotypical or morphological adaptations in *A*. *bangokensis*. Further identification in embryonic and/or early developmental stage should be expanded in future study, as we analyzed the transcriptome of adult *A*. *bangokensis*.

**Table 3:** List of eye-related genes identified in the *Allobathynella bangokensis* transcriptome, presented with RPKM values and matching species information. The genes and reference protein IDs of *Daphnia pulex* were adopted from a previous study [37].

Gene family	Reference protein ID (*Daphnia pulex*)	Matched sequence ID of *A*. *bangokensis*	RPKM	Matched species	E-value	Accession No.
***Visual system specification gene family***						
Decapentaplegic	*Dappu-347232*	-	-	-	-	-
Engrailed (En)	*Dappu-290630*	Allo_10518	8.43	*Pholcus phalangioides*	1.00E-43	CYF18415
	*Dappu-290638*	-	-	-	-	-
Hedgehog (Hh)	*Dappu-347555*	Allo_11417	10.35	Daphnia pulex*	3.00E-07	EFX65836
Wnt1	*Dappu-44743*	-		-	-	-
***Retinal determination network gene family***						
Dachshund (Dac)	*Dappu- 310049*	-	-	-	-	-
Eyes-absent (Eya)	*Dappu- 204955*	-	-	-	-	-
Eyegone (Eyg/Toe)	*Dappu- 253988*	-	-	-	-	-
Pax-6	*Dappu- 249978*	Allo_67774	4.98	*Parasteatoda tepidariorum*	1.00E-79	XP_015917132
	*Dappu-249991*	-	-	-	-	-
Six 1/2	*Dappu-65962*	Allo_28121	2.71	Pediculus humanus corporis**	2.00E-89	XP_002431193
***Photoreceptor differentiation gene family***						
Epidermal Growth Factor Receptor (EGFR)	*Dappu-324147*	Allo_53476	1.90	Halyomorpha halys**	0	XP_014284376
	*Dappu- 321139*	Allo_40048	27.72	Daphnia magna*	0	KZS10602
Kruppel (Kr)	*Dappu-290527*	Allo_38368	7.75	Strigamia maritima**	2.00E-30	AAY45764
Glass (Gl)	*Dappu-234903*	-	-	-	-	-
Notch	*Dappu-328760*	Allo_53041	27.09	Parhyale hawaiensis*	0	ABK56706
Spitz (Spi)	*Dappu-271304*	-	-	-	-	-
CVC Homeobox (Vsx)	*Dappu-323346*	-	-	-	-	-
***Phototransduction gene family***						
Arrestin (Arr)	*Dappu-216585*	Allo_38980	10.80	Marsupenaeus japonicas*	0	AME17864
	*Dappu-207575*	-	-	-	-	-
Gq-alpha	*Dappu-211929*	Allo_36916	0.44	Marsupenaeus japonicas*	0	BAH98115
	*Dappu-188187*	-	-	-	-	-
Phospholipase-C (PLC)	*Dappu- 226357*	Allo_48820	9.30	Homarus americanus*	1.00E-99	AAD32609
	*Dappu- 304714*	-	-	-	-	-
Transient Receptor Potential Channel (TRPC)	*Dappu-54362*	Allo_59564	0.37	Daphnia magna*	9.00E-13	EFX75016
	*Dappu- 309057*	Allo_30981	10.46	Tribolium castaneum**	3.00E-52	KYB28200

* Crustacea

** Insecta

Since stygofauna have reduced or absent eyes and have enhanced non-optic sense organs without pigmentation [44, 45], the absence of pigments and eyes is observed in *A*. *bangokensis*. A total of 25 and 26 eye pigmentation genes that were retrieved from *Drosophila melanogaster* and *Tribolium castaneum* respectively [46], were also aligned to the entire transcripts of *A*. *bangokensis*, but there was no matched transcripts in *A*. *bangokensis* transcriptome. Although mRNA expression of putative opsin genes was not observed in the *A*. *bangokensis* transcriptome, it will be interesting to investigate the presence or absence of the opsin repertoire in the genome and the evolutionary development of alternative sensory organs in future studies. Several previous examples showed the potential correlation between reduced or absent eyes and transcriptional expression of the opsin repertoire. No loss of gene function in opsin gene paralogs with a reduced level of gene expression was reported in the cave-adapted amphipod *Gammarus minus* [36]. The authors suggested that loss of expression of opsin genes without loss of gene function is explained by the pleiotropic roles of opsin genes [36]. Extensive transcriptomic analysis revealed that three independently evolved subterranean diving beetles lack transcripts of nearly all opsin photoreceptor genes, whereas the two surface beetles showed evidence of transcriptional expression of a full suite of insect visual and non-visual opsin genes [47]. Thus, research on the absence or presence of putative opsin genes from the *A*. *bangokensis* genome will be useful to understand the regressive evolution of eye reduction in the Bathynellacea lineage.

### Heat shock protein superfamily

**Fig 3:**
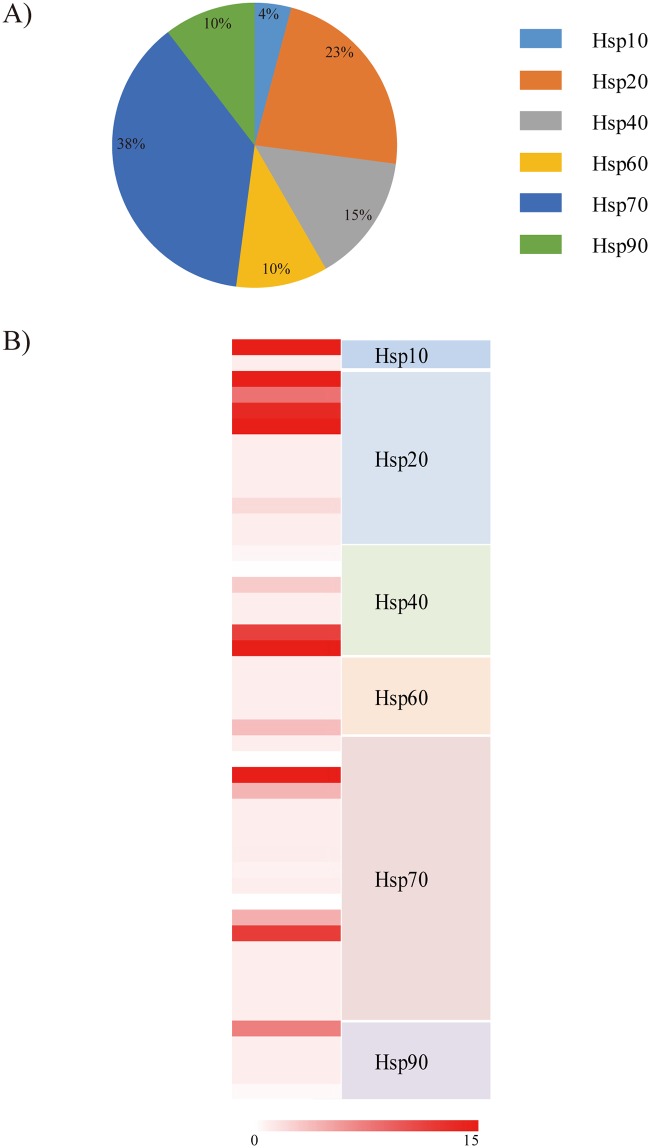
Analysis of heat shock protein superfamily of the *Allobathynella bangokensis*. The compositions and percentage rankings of the *Allobathynella bangokensis* heat shock protein (*Hsp*) superfamily (A) and their basal transcript levels represented by RPKM values (B). Detailed information is appended in the supplementary materials (S8 Table).

Environmental changes or stress factors induce molecular and systemic metabolism to maintain cellular and physiological homeostasis. The heat shock protein (*Hsp*) superfamily is the most conserved protein present in both prokaryotes and eukaryotes. In general, their expression can be induced by a wide variety of physiological and environmental stimuli [48]. Proteins of the *Hsp* superfamily function as molecular chaperones and are the key components responsible for assisting the correct folding of nascent or stress-accumulated misfolded proteins and for preventing aggregation [49]. In the *A*. *bangokensis* transcriptome, 48 *Hsp* genes distributed among five subfamilies (i.e., *Hsp10*, *Hsp20*, *Hsp40*, *Hsp60*, *Hsp70*, and *Hsp90*) were annotated (Fig 3A; S8 Table). The diversity of the *A*. *bangokensis Hsp* family was unpredictable, since a drastic reduction in the ranges of daily and annual temperatures is a distinguishing characteristic of the subterranean zone [50]. Overall, the RPKM values for most *Hsp* genes were relatively low; 31 *Hsp* genes (65%) showed RPKM values < 1 (Fig 3B; S8 Table). Some *Hsp* genes are expressed constitutively at minimal or basal levels, while other *Hsp* genes can be induced rapidly in response to environmental stimuli [51]. Thus, we expect that *A*. *bangokensis* employs an effective holding and folding defense system using a combination of differentially expressed *Hsp* genes at low levels. This defense system diminished cellular stress, which can be triggered by even small changes in subterranean environmental conditions.

Regardless of their chaperonin function, a previous report highlighted a distinct gain of function of the *hsp90α* gene in the cavefish *Astyanax mexicanus* [52]. *hsp90α* is expressed specifically in the cavefish lens starting just prior to apoptosis, while the expression was not observed in the lens of eyed surface-dwelling *A*. *mexicanus* (surface fish), suggesting that activation of the *hsp90α* gene may be required for eye degeneration and apoptosis in the lens in cavefish [52]. Therefore, further study of *Hsp* function in *A*. *bangokensis* is needed to understand its phenotypic adaptation. Also, it has been widely suggested that *Hsp* genes are good biomarkers for numerous environmental changes [48, 49]. Sequence information of the *A*. *bangokensis Hsp* family can be applied to subterranean environmental monitoring by analyzing mRNA and protein expression profiles after induction by environmental stressors.

### Innate immune system

In general, metazoans have an innate immune system, which consists of cellular and humoral metabolism, as the first line of defense against pathogenic bacteria, fungi, viruses, and metazoan parasites using recognition, regulation, and response [53, 54]. Many key pathways employing genes involved in innate immunity are conserved between mammals and arthropods including crustaceans [53, 55]. In the *A*. *bangokensis* transcriptome, we found conservation of most immunity-related gene families previously analyzed in arthropods [55–58]. As shown in Table 4, a variety of innate immunity-related sequences corresponding to adhesive proteins, antimicrobial proteins, apoptosis- and cell cycle-related proteins, cellular defense effectors, immune regulators, pattern recognition proteins, proteases, protease inhibitors, reduction/oxidation-related proteins, signal transduction-related proteins, and stress proteins were observed. These results suggest that complex immune-relevant gene sets are actively expressed to maintain cellular homeostasis even in subterranean animals. Immune-relevant gene sets will help extend our knowledge on the immune systems of syncarids in comparative aspects and ecological genetics within Arthropoda.

**Table 4:** Immune-relevant genes annotated in the *Allobathynella bangokensis* transcriptome database.

Seq ID	Gene name	Matched species	E-value	Accession No.	RPKM
***Adhesive protein***					
Allo_01147	Cadherin 87	Cephus cinctus**	8.00E-07	XP_015588457	9.55
Allo_18151	Galectin	Litopenaeus vannamei*	3.00E-29	AGV04659	4.61
Allo_40297	multiple PDZ domain protein isoform X2	Acyrthosiphon pisum**	1.00E-147	XP_008181772	20.81
Allo_74308	Tetraspanin-5-like isoform 1	Athalia rosae**	7.00E-152	XP_012258733	14.26
Allo_63960	Transglutaminase	Pacifastacus leniusculus*	7.00E-47	AAK69205	4.53
Allo_31604	Histone H2A-like	Diuraphis noxia**	2.00E-68	XP_015363720	26.19
Allo_69601	Histone H3.3	*Oncorhynchus mykiss*	1.00E-41	ACO08041	8.80
Allo_29964	integral membrane,	*Ixodes scapularis*	1.00E-68	XP_002412964	0.79
***Apoptosis and cell cycle***					
Allo_61561	inhibitor of apoptosis protein	Penaeus monodon*	3.00E-166	ABO38431	0.00
Allo_68354	β-catenin-like protein 1	*Limulus polyphemus*	1.00E-167	XP_013791309	0.70
Allo_12816	Catenin delta-2	Lasius niger**	2.00E-56	KMQ88737	0.74
Allo_14751	Programmed cell death protein 4	Zootermopsis nevadensis**	2.00E-146	KDR08650	38.58
Allo_56958	Rab-1	Macrobrachium rosenbergii*	7.00E-104	AJC97112	0.77
Allo_55831	ras-related Rab-32B-like	*Aplysia californica*	3.00E-60	XP_005097933	0.32
***Cellular defense effecter***					
Allo_70113	Dual oxidase	Marsupenaeus japonicas*	0.0	BAM76968	12.50
Allo_25381	fibrinogen-like protein	Fenneropenaeus merguiensis*	9.00E-58	AKR15662	0.00
Allo_27844	Thioredoxin reductase 3	*Crassostrea gigas*	0.0	XP_011419860	15.24
Allo_56333	c-X-C motif chemokine 15-like	*Bos taurus*	1.00E-10	XP_003582386	2.95
***Immune regulator***					
Allo_24131	signal sequence receptor beta-like protein	*Plectreurys tristis*	8.00E-59	AJD25284	16.15
Allo_72274	Translocon-associated protein subunit α	Zootermopsis nevadensis**	2.00E-73	KDR17543	36.74
Allo_00305	Carboxypeptidase B2	Daphnia magna*	5.00E-134	KZS09622	82.73
***Protease and protease inhibitor***					
Allo_47130	26S protease regulatory subunit 10B X2	*Myotis lucifugus*	5.00E-135	XP_014318322	2.01
Allo_23607	α-2-macroglobulin 2 isoform 3	Pacifastacus leniusculus*	0.0	AEC50083	0.64
Allo_40857	trypsin	Litopenaeus vannamei*	6.00E-112	CAA75311	0.00
Allo_30909	Cystatin 2	Cherax quadricarinatus*	7.00E-141	ALC79585	0.00
Allo_37572	Cathepsin A	Eriocheir sinensis*	0.0	ADO65982	14.52
Allo_54726	Cathepsin B	Fenneropenaeus chinensis*	1.00E-175	AHA83423	1.27
Allo_56730	Cathepsin C	Fenneropenaeus chinensis*	3.00E-122	ACG60902	0.00
Allo_64644	Cathepsin D	Palaemon carinicauda*	0.0	AGJ03549	33.05
Allo_54848	Cathepsin L protein	Cherax quadricarinatus*	0.0	AJS13771	0.00
Allo_54759	inter-α-trypsin inhibitor heavy chain H6	*Monodelphis domestica*	1.00E-14	XP_016282619	6.49
Allo_54846	matrix metalloproteinase-14	Papilio Xuthus**	1.00E-173	XP_013166430	5.18
Allo_29726	protein phosphatase	Fenneropenaeus chinensis*	0.0	AHE40944	34.04
Allo_00427	serine carboxypeptidase CPVL-like	*Neolamprologus brichardi*	1.00E-159	XP_006801474	26.79
***Redox***					
Allo_39210	hypothetical ferritin light-chain subunit	Rimicaris exoculata*	5.00E-41	ACR43472	0.44
Allo_37530	Peroxiredoxin	Penaeus monodon*	1.00E-129	ABZ80828	52.99
Allo_23907	selenium-binding protein 1-A-like	*Corvus brachyrhynchos*	1.00E-126	XP_008627838	14.72
***Signal transduction***					
Allo_28397	calmodulin-A-like isoform X1	Microplitis demolitor**	9.00E-68	XP_008550049	6.82
Allo_50919	cAMP-dependent kinase	Nasonia vitripennis**	4.00E-16	ACH99585	1.20
Allo_69397	Casein kinase II subunit beta	Cerapachys biroi**	4.00E-133	XP_011340131	8.24
Allo_55642	COP9 signalosome complex subunit 2	Athalia rosae**	0.0	XP_012264747	20.29
Allo_14758	mitogen-activated protein kinase	Fenneropenaeus chinensis*	2.00E-131	AHA83424	12.99
Allo_20185	tyrosine- kinase Src64B-like isoform 1	Zootermopsis nevadensis**	0.0	KDR17588	0.00
Allo_04375	Protein vav	Neodiprion lecontei**	2.00E-34	XP_015521070	0.34
***Stress protein***					
Allo_50356	ATP synthase F0 subunit 6	*Aradacanthia heissi*	8.00E-30	ADQ64032	12.38
Allo_22874	heat shock factor binding 1-like	Trachymyrmex zeteki**	3.00E-22	KYQ58790	2.21
Allo_40206	melanoma-associated antigen G1	*Myotis davidii*	2.00E-08	XP_006771818	3.54
Allo_33230	CD63 antigen	Lepeophtheirus salmonis*	3.00E-61	ACO12374	10.02
Allo_21482	B-cell lymphoma leukemia 11B-like	Zootermopsis nevadensis**	2.00E-05	KDR23016	0.69
Allo_23197	Transferrin	Zootermopsis nevadensis**	4.00E-144	KDR19744	0.00
Allo_21081	ubiquitin specific peptidase 25	*Astyanax mexicanus*	6.00E-17	XP_007244811	6.83
Allo_26367	LITAF	Litopenaeus vannamei*	2.00E-25	AEK86526	4.32
***Acute phase response/inflammation***					
Allo_05913	heme oxygenase 1-like isoform X2	Apis dorsata**	1.00E-06	XP_006620087	5.49

* Crustacea

** Insecta

In arthropods, pathogens are selectively recognized by major signaling pathways, such as Toll, immune deficiency (IMD), and Janus kinase (JAK)-signal transducer of activators of transcription (STAT) for activation of immune effectors [57]. Since there is no information on the intactness of the major pathways in subterranean crustaceans, we evaluated the absence or presence of pathways using *in silico* approaches (e.g., domain analysis, phylogenetic analysis) based on KEGG pathway analysis of the *A*. *bangokensis* transcriptome. Detailed protein interactions and their functions for all members involved in each pathway are mostly omitted in this manuscript, as several valuable publications have extensively reviewed their roles in arthropods [56–60]. The identified genes and information pertaining to the absence or presence of expression, sequence IDs, matching species, E-values, and GenBank accession numbers are presented in Supplementary material (S9 Table).

**Fig 4:**
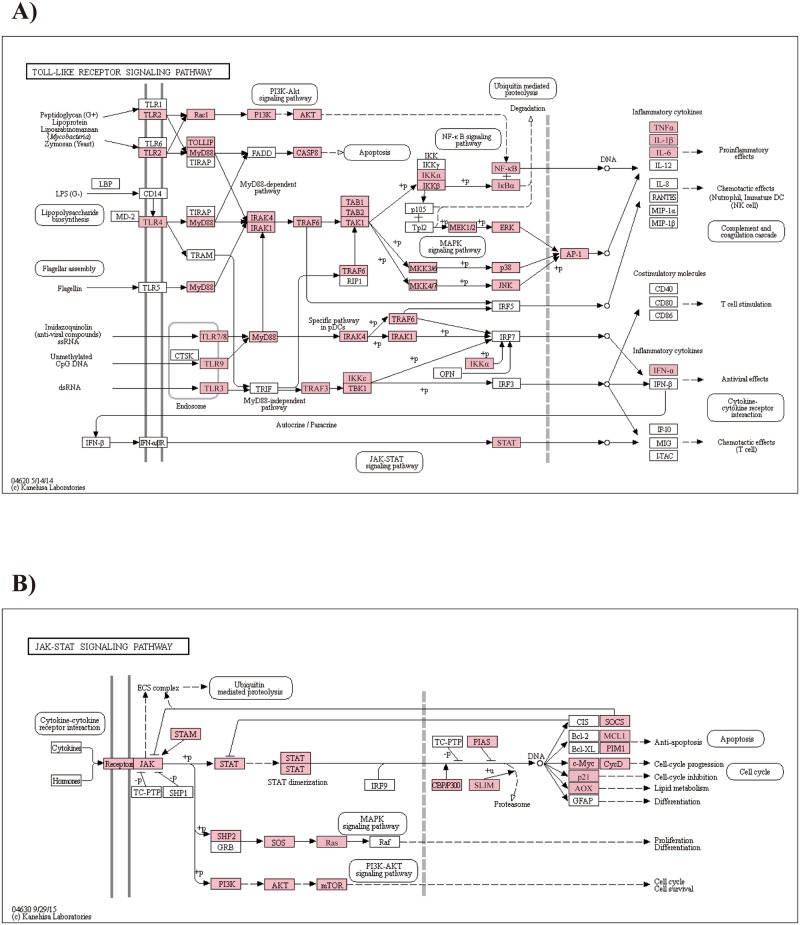
Toll-like receptor and JAK-STAT signaling pathway. *Allobathynella bangokensis* transcripts coding for the corresponding enzymes of the A) Toll-like receptor and B) JAK-STAT signaling pathways from the KEGG database. The annotated enzymes are shown in pink boxes.

Of the pattern recognition receptors, the Toll-like receptors (TLRs) represent an evolutionarily conserved host defense mechanism in both invertebrates and vertebrates [61]. Based on the type of adaptor molecule, individual TLR signaling pathways differentially activate downstream components in the signaling cascade, causing activation of the transcription factor NF-κB and interferon (IFN) regulatory factors [62]. Although there are no reports regarding MyD88-independent pathways in invertebrates, crustacean *TLRs* and *MyD88* are transcriptionally responsive to immune stimulation [63–67]. We found all putative homologs of TLR signaling pathway members to be highly conserved across arthropods, including Spätzle cytokine (Allo_71790), MyD88 (Allo_03624), Pelle (interleukin-1 receptor-associated kinase 1, IRAK1, Allo_70004), Tube (IRAK4, Allo_32335), Pelle-interacting protein Pellino (Allo_43963), Toll-interacting protein (TOLLIP, Allo_08036), sterile alpha- and armadillo-motif-containing protein (SARM, Allo_67175), evolutionarily conserved signaling intermediate in the Toll pathway (ECSIT, Allo_69618), tumor necrosis factor receptor-associated factor (TRAF, Allo_61987), Cactin (Allo_58700), NF-κB inhibitor Cactus (IκB, Allo_32054), and Dorsal (Rel/NF-κB, Allo_30610) (Fig 4A; S9 Table). Thus, the Toll pathway appears to be intact in *A*. *bangokensis*.

The JAK-STAT signaling pathway is evolutionarily conserved and mediates the response to chemical messenger molecules such as diverse cytokines, IFNs, growth factors, and related molecules [68]. In the *A*. *bangokensis* transcriptome, putative homologs of the transmembrane cytokine receptor Domeless (Allo_26652), JAK (Allo_51891), STAT (Allo_07305), signal transducing adaptor molecule (Allo_52924), suppressor of cytokine signaling (Allo_16562), and protein inhibitors of activated STAT (PIAS, Allo_52571) were all identified by KEGG analysis as commonly observed key components in crustaceans (Fig 4B; S9 Table) [60]. The transcriptional presence of key modulators of the JAK-STAT pathway, as well as downstream components of the pathway, suggests that the JAK-STAT pathway is strongly involved in the regulation of *A*. *bangokensis* immunity.

Similar to the JAK-STAT pathway, many downstream members of the IMD signaling pathway are also conserved in the *A*. *bangokensis* transcriptome. The IMD pathway is activated mainly by Gram-negative bacteria and is separated into the NF-κB/Relish and Jun N-terminal kinase (JNK) branches [69]. Recently, Rosa et al [60] summarized 23 key component proteins of the insect IMD pathway, 17 of which are commonly identified in crustaceans. Since the KEGG database does not include the IMD pathway, manual identification was employed in the *A*. *bangokensis* transcriptome, which showed that most of the components of the IMD pathway have a homologue in the *A*. *bangokensis* transcriptome. Among the 17 crucial proteins involved in the crustacean IMD pathway, 16 were annotated in the *A*. *bangokensis* transcriptome (S9 Table), and only defense repressor 1 was not identified at the transcriptional level. These 16 proteins are IMD (Allo_52952), enzymes involved in ubiquitination (UEV1a, Allo_70733; Effete/Ubc13, Allo_20821; Bendless/Ubc5, Allo_28290), the negative regulators Caspar (Fas-associated factor 1, Allo_72340) and POSH (E3 ligase Plenty of SH3, Allo_27226), inhibitor of apoptosis IAP2 (Allo_73002), transforming growth factor-β-activated kinase 1 (TAK1, Allo_01961), TAK1-binding protein 2 (TAB2, Allo_72573), IκB kinase IKK-α (Allo_18196), a Relish-like Rel/NF-κB transcription factor (Allo_40693), Caudal homeobox protein (Allo_52669), mitogen-activated protein kinase kinase Hemipterous (Allo_31075), the JNK homolog Basket (Allo_17845), the negative regulator Puckered (Allo_58869), Activator protein 1, and the transcription factor Jun-related antigen (Allo_32187). Overall, the TAK1/TAB2 complex-activated JNK pathway was apparently conserved in *A*. *bangokensis*, but several members involved in the NF-kB/Relish branch were missing from the transcriptome database.

In crustaceans, the absence or presence of several components of the IMD pathway (i.e., peptidoglycan recognition proteins, Fas associated protein with death domain (FADD), death related ced-3/Nedd2-like (DREDD), poor IMD response upon knock-in (Pirk), IκB kinase Kenny/NEMO, Fos-related antigen/Kayak (Fra)) is controversial [60], and *A*. *bangokensis* also did not express FADD, DREDD, or Pirk at the transcriptional level. Thus, this result supports that *A*. *bangokensis* lacks several crucial members of a well-conserved IMD pathway as shown by the missing sequences in Insecta (e.g., Hemiptera), Crustacea, and Chelicerata [60]. Further experiments are needed to clarify the absence/presence of these genes at the genome level. However, Kenny/NEMO (Allo_64535) and Fra (Allo_55867) contigs were observed in the database. Taken together, we have confirmed that major immune responsive pathways, such as Toll, JAK-STAT, and the JNK branch of IMD pathway, involved in the innate immune system of *A*. *bangokensis* are evolutionarily conserved across crustaceans at the transcriptional level.

### Antioxidant defense system

**Fig 5:**
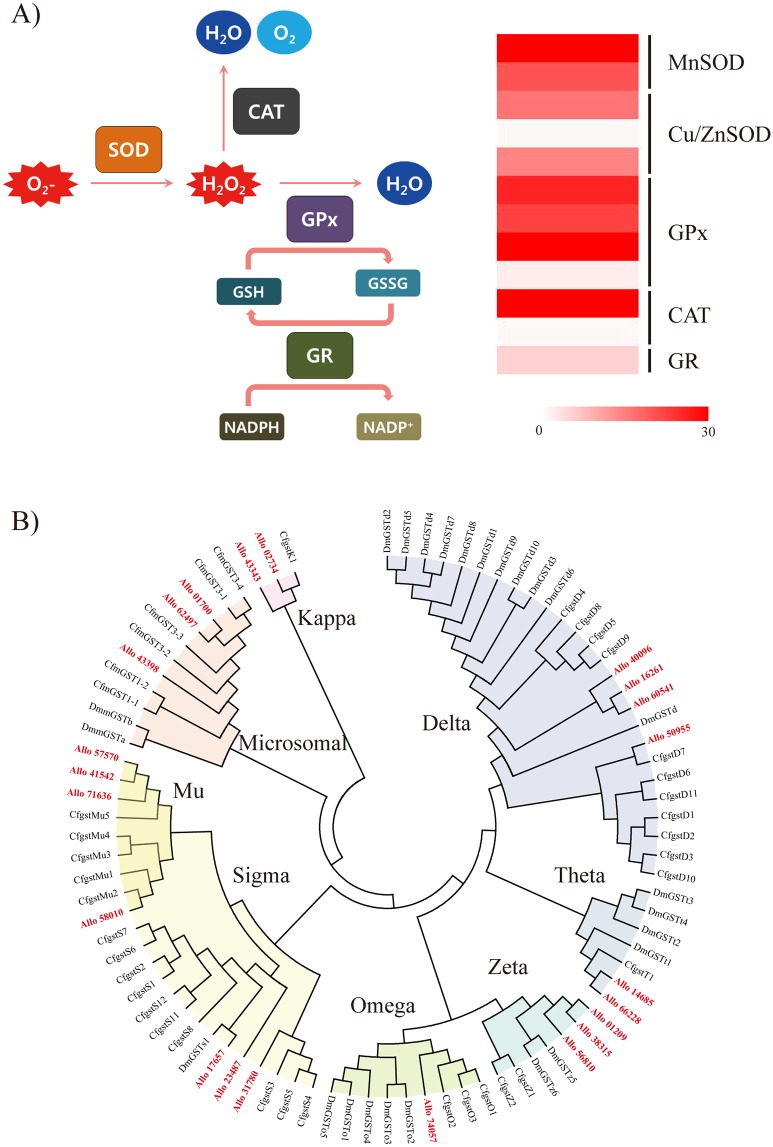
Antioxidant defense system in *Allobathynella bangokensis*. A) Schematic diagram representing a proposed cascade for the antioxidant defense system, along with RPKM values of the *Allobathynella bangokensis* transcripts coding for the corresponding enzymes in this system. B) Phylogenetic tree of glutathione *S*-transferase (GST) proteins from *A*. *bangokensis* and from arthropods constructed by the Bayesian method.

Reactive oxygen species (ROS) such as superoxide anion, hydrogen peroxide, and hydroxyl radical are generated during mitochondrial oxidative phosphorylation and during the cellular response to xenobiotics, cytokines, and bacterial invasion [70]. Oxidative stress results in direct or indirect ROS-mediated damage to nucleic acids, proteins, and lipids. To maintain cellular homeostasis, antioxidant defense systems composed of catalase, glutathione depletion, glutathione reductase, glutathione peroxidase, and superoxide dismutase (SOD) can be induced to eliminate excess ROS levels. Based on the annotation results, we found that *A*. *bangokensis* has an antioxidant defense system similar to those of other arthropods (Fig 5A). In addition, the basal RPKM values of all genes indicated relatively high transcription levels, and thus it remains to be determined how *A*. *bangokensis* protects its cells and bodies from exogenous oxidative stress.

Glutathione *S*-transferases (GSTs) and their isoforms play important roles in the cellular antioxidant defense system. In addition to their activities under oxidative stress conditions, GST family members play crucial roles in drug metabolism and the detoxification pathway. Phase I, phase II, and phase III detoxification systems are an efficient means of protecting against the potential impacts via both metabolic homeostasis and elimination of exogenous molecules (e.g., xenobiotics) in animals [71]. Of these detoxification systems, phase II is characterized by reductive or conjugative modification reactions of phase I metabolites to endogenous compounds through GST enzymatic activity. Thus, GSTs and their isoforms have been commonly used as strong biomarkers of both oxidative stress and cellular toxicity. In the *A*. *bangokensis* transcriptome, 22 *GST* genes were annotated and grouped into eight well-characterized GST subfamilies (Delta, Kappa, Mu, Omega, Sigma, Theta, Zeta, and microsomal GST) of arthropods (Fig 5B). Taken together, we provide evidence for a robust antioxidant defense system in *A*. *bangokensis* and maintenance of cellular homeostasis in subterranean crustaceans. Elucidating the mechanisms underlying activation of the antioxidant defense system by environmental changes is crucial for the development of effective monitoring strategies for subterranean ecosystems.

## Conclusions

The ability of *A*. *bangokensis* to survive in such extreme subterranean environments suggests that they have evolved adaptations by employing molecular homeostatic mechanisms. The relationships between animals and their hyporheic/groundwater environments are being investigated continuously, and transcriptomic sequencing of *A*. *bangokensis*, as a sentinel species, renders it an optimal model for studying molecular adaptation and response mechanisms to harsh environmental conditions. Although the transcriptional responses induced by environmental changes or the unique adaptive metabolism of *A*. *bangokensis* are not discussed in this study due to the limited samples from subterranean regions, the transcriptomic database and gene composition provide the basis for clarifying the adaptive and responsive metabolic pathways. Thus, further identification and confirmation of the functions of conserved genes or pathways as well as the dissection of the genetic architecture of response genes will be useful for the study of adaptive mechanisms in subterranean ecosystems.

## Supporting Information

S1 Fig General habitus (lateral) of *Allobathynella bangokensis* Park and Cho, 2016. (TIF)

S2 Fig The compositions and percentage rankings of the most abundant InterPro domains from the InterProScan annotation of *Allobathynella bangokensis* transcript contigs. (TIF)

S3 Fig Gene ontology (GO) analysis of *Allobathynella bangokensis* transcript contigs in terms of A) biological processes, B) molecular functions, and C) cellular components. Detailed information is appended in S3–S5 Tables.
(TIF)

S1 Table Brief summary of annotation. (XLSX)

S2 Table Blast top hit species. (XLSX)

S3 Table Gene ontology (GO) analysis of *A*. *bangokensis* transcript contigs in terms of biological processes. (XLSX)

S4 Table Gene ontology (GO) analysis of *A*. *bangokensis* transcript contigs in terms of molecular functions. (XLSX)

S5 Table Gene ontology (GO) analysis of *A*. *bangokensis* transcript contigs in terms of cellular components. (XLSX)

S6 Table KEGG analysis of the *A*. *bangokensis* transcriptome. (XLSX)

S7 Table List of opsin genes of other species. (XLSX)

S8 Table List of *Hsp* genes of *A*. *bangokensis*. (XLSX)

S9 Table List of Immune-relevant genes of *A*. *bangokensis*. (XLSX)
