# Supplementary figures and images for: First Insights into the Subterranean Crustacean Bathynellacea Transcriptome: Transcriptionally Reduced Opsin Repertoire and Evidence of Conserved Homeostasis Regulatory Mechanisms
Source: PLoS One. 2017 Jan 20;12(1):e0170424. doi: 10.1371/journal.pone.0170424 (PMC5249073; doi:10.1371/journal.pone.0170424)

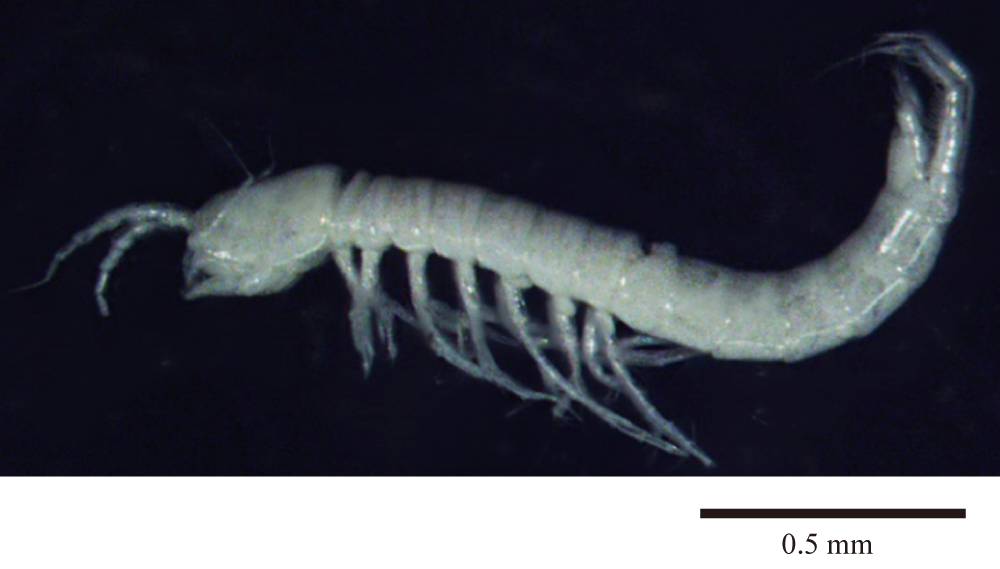

Supplement: S1 Fig — (TIF) [file pone.0170424.s001.tif]

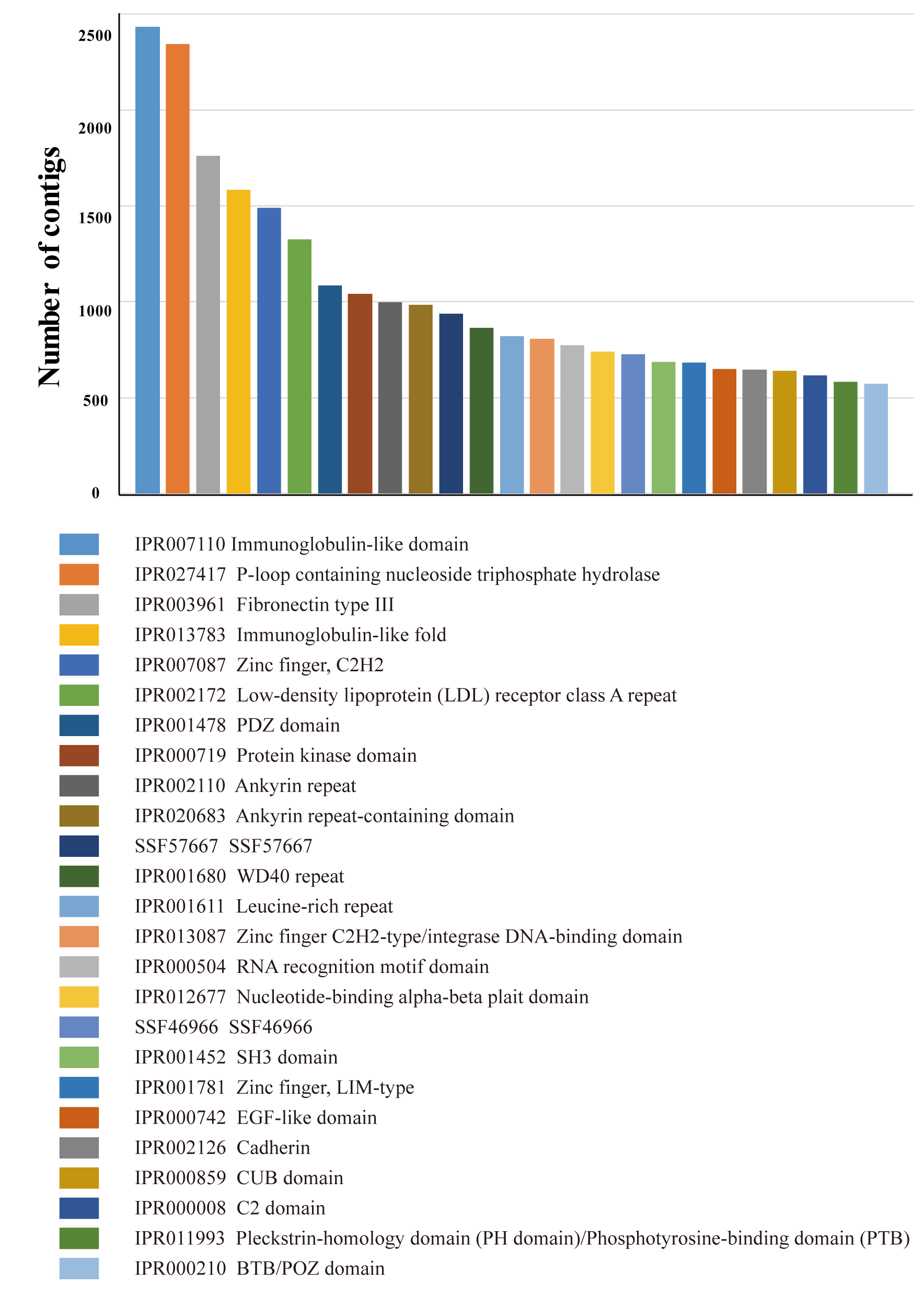

Supplement: S2 Fig — (TIF) [file pone.0170424.s002.tif]

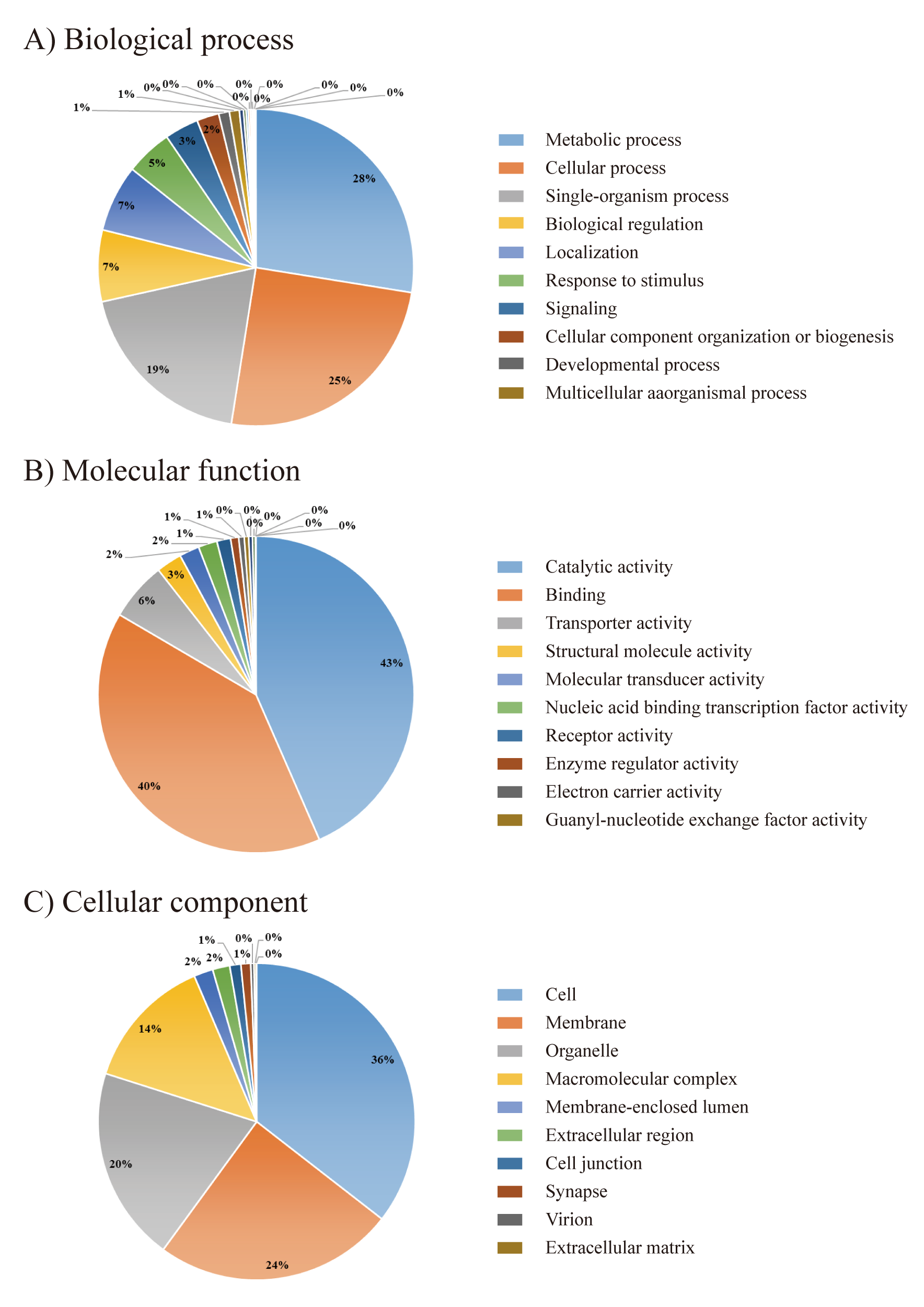

Supplement: S3 Fig — Detailed information is appended in S3–S5 Tables. (TIF) [file pone.0170424.s003.tif]
